# Evaluation of serum retinol-binding protein-4 levels as a biomarker of poor short-term prognosis in ischemic stroke

**DOI:** 10.1042/BSR20180786

**Published:** 2018-09-12

**Authors:** Yan-yan Zhu, Jian-long Zhang, Li Liu, Yingbo Han, Xiaomin Ge, Shuai Zhao

**Affiliations:** Department of Neurology, The First Affiliated Hospital of Xinxiang Medical University, Xinxiang, China

**Keywords:** ischemic stroke, prognosiS, Retinol-binding protein 4, vitamin A

## Abstract

The aim was to investigate the relationship between retinol-binding protein 4 (RBP4) levels and short-term functional outcome, and to determine its possible role in acute ischemic stroke (AIS). In a prospective observational study, 299 first-ever AIS who were admitted to our hospital were included. Serum levels of RBP4 were assayed and severity of stroke was evaluated with the National Institutes of Health Stroke Scale (NIHSS) score on admission. The prognostic value of RBP4 to predict the poor outcome within 3 months was compared with the NIHSS and with other known outcome predictors. The median age of the included patients was 66 (interquartile range (IQR): 55–77) years and 155 (51.8%) were women. A poor functional outcome was found in 88 patients (29.4%), and significantly higher RBP4 values were found in poor outcomes rather than good outcomes patients (*P*<0.001). The poor outcomes distribution across the RBP4 quartiles ranged between 9.3% (first quartile) and 60.8% (fourth quartile). In multivariate models comparing the second(Q2), third, and fourth quartiles against the first quartile of the RBP4, RBP4 in Q3 and Q4 were associated with poor functional outcome, and increased risk of poor functional outcome by 144% (OR: 2.44; 95% confidence interval (CI): 1.22–5.03) and 602% (7.02; 3.11–12.24), respectively. Interestingly, RBP4 improved the NIHSS score (area under the curve (AUC) of the combined model, 0.79; 95% CI: 0.74–0.85; *P*<0.001). The data showed that elevated serum levels of RBP4 at admission were associated with severity and prognosis of AIS, suggesting that vitamin A metabolism or impaired insulin signaling could be involved.

## Introduction

Plasma retinol-binding protein 4 (RBP4) is the 21-kDa transporter of all-*trans* retinol that circulates in plasma as a moderately tight 1:1 molar complex of the vitamin with the protein [1]. This protein belongs to the lipocalin family and is the specific carrier for retinol (vitamin A) in the blood [2].

Previous studies had suggested that the association of circulating levels of RBP4, a plasma retinol transporter and adipokine, with traditional (e.g. blood pressure) and non-traditional cardiovascular risk factors (e.g. cytokines) mainly through the impairment of glucose and lipid metabolism and adipose tissue dysfunction [3]. Circulating levels of RBP4 were found to correlate with insulin resistance [4], impaired glucose tolerance and type 2 diabetes [5,6], and metabolic syndrome [7]; subsequent studies also suggested that the associations of RBP4 with cardiovascular disease (CVD, [8]). It has recently been suggested that inflammation produced by RBP4 induces insulin resistance and CVD [9]. Although, the association of RBP4 with an increased risk of CVD has been proposed [10], its prognostic value in carotid [11] or coronary [12] atherosclerosis progression still lacks consensus.

Stroke is the second leading cause of death and the leading cause of serious, long-term disability worldwide [13]. Hypertension [14] and atherosclerosis [15] were common risk factors for stroke. Interestingly, previous study had showed that serum levels of RBP4 were associated with blood pressure [16] and carotid atherosclerosis [17]. Furthermore, Llombart et al. [18] suggested that RBP4 might be useful as diagnostic biomarkers to differentiate ischemic stroke and intracerebral hemorrhage. Therefore, we speculated that serum RBP4 might be associated with stroke risk and prognosis. In the present study, we sought to investigate the relationship between RBP4 levels and short-term functional outcome, and to determine its possible role in acute ischemic stroke (AIS).

## Materials and methods

### Patients and study design

This was a prospective cohort study at Department of Emergency in our hospital. From January 2016 to December 2017, all patients with first-ever AIS were included. All patients were admitted within 24 h of experiencing a new focal or global neurological event. A baseline MRI scan was performed in all patients at admission to verify the diagnosis. The patients with the following criteria were excluded: (i) malignant tumor, (ii) and a history of recent surgery or trauma during the preceding 2 months; (iii) liver and kidney function insufficiency; (iv) acute and chronic inflammation; metabolic abnormalities (diabetes not included), (v) febrile disorders and autoimmune diseases; (vi) other neurological diseases (cerebral hemorrhage, Parkinson’s disease, and Alzheimer’s disease).

In the present study, 150 age and gender-matched healthy volunteers were assigned to the normal group. The median age of control cases was 65 (interquartile range (IQR): 55–77) years and 52% were women. The study was approved by the Ethics Committee of First Affiliated Hospital of Xinxiang Medical University The patients or their relatives gave written informed consent prior to entering the study.

### Clinical variables and neuroimaging

At baseline, demographical and clinical data including: age, sex, body mass index (BMI), temperature, systolic blood pressure, conventional vascular risk factors (hypertension, diabetes mellitus, atrial fibrillation, coronary heart disease, smoking habit, and family history for stroke and/or CVD) were obtained. In addition, the information about pre-stroke therapy (oral anticoagulants or antiplatelet agents) and acute treatment (IV thrombolysis and/or mechanical thrombectomy) were also recorded. All patients received treatment according to current guidelines. Stroke severity was assessed on admission using the National Institutes of Health Stroke Scale (NIHSS, range from 0 to 42) by a neurologist [19]. Stroke subtype and the clinical stroke syndrome were classified according to TOAST (Trial of Org 10172 in Acute Stroke Treatment) criteria [20] and the Oxfordshire Community Stroke Project (total anterior circulation syndrome (TACS); partial anterior circulation syndrome (PACS); lacunar syndrome (LACS); and posterior circulation syndrome (POCS)) [21], respectively. Brain imaging (MRI) was performed routinely within 24 hours of admission. Diagnosis of stroke was based on the results of strict neurological examination (MRI) according to the International Classification of Diseases, ninth revision. MRI was performed using a stroke protocol (T1-, T2-, and diffusion-weighted imaging (DWI) sequences and a magnetic resonance angiography). DWI lesion volumes were calculated by an experienced neurologist (X.G.) unaware of the clinical and laboratory information according to the formula 0.5 × a × b × c (a = the maximal longitudinal diameter; b = the maximal transverse diameter perpendicular to a; c = the number of 10-mm slices containing infarct) [22].

### End points and follow-up

The functional outcome was determined in month 3 according to the modified Rankin Scale (mRS, range from 0 to 6) [23] blinded to laboratory information. The primary end point was functional outcome, which was divided into poor functional outcome (defined as mRS score of 3–6 points) and good functional outcome (mRS: 0–2) according to mRS. Secondary end point was death or withdrawn from any cause within a 3-month follow-up. Outcome assessment was performed by a trained medical staff (Y.H.) with a structured follow-up telephone interview with the patient or, if not possible, with the relative.

### Laboratory analyses

Fasting serum samples were drawn from the antecubital vein at the first morning after admission and within 48 h of symptom onset (within 0–6 (*n*=73), 6–12 (*n*=98), 12–24 (*n*=92), and 24–48 (*n*=36) h from symptom onset). After centrifugation, the serum samples were immediately stored at –80°C before assay. The information about time from onset to blood collection was recorded. Concentrations of serum RBP4 were batch analyzed using a commercially available Quantikine® ELISA kit for human RBP4 (Cat#DRB400; R&D Systems, Minneapolis, MN). The lower detection limit for RBP4 was preset as 0.60 ng/ml and the detection range was 1.6–100 ng/ml. Inter-assay and intra-assay coefficients of variation were 5.0–9.0% and 4.0–7.5%, respectively. Due to the high levels of RBP4 (μg/ml) in the blood sample, 1:100 dilution was used. Other blood biomarkers, for instance triglyceride, cholesterol, high-density lipoprotein (HDL), low-density lipoprotein (LDL), high-sensitivity C-reactive protein (Hs-CRP), fasting blood glucose (FBG) were tested using an enzyme cycling method by BS-2000M (Mindray, Shenzhen, China). All those tests were done in duplicates and samples with a CV exceeding 10% were reanalyzed.

### Statistical analysis

Results are expressed as percentages for categorical variables and as medians (quartiles) for the continuous variables. Spearman’s rank correlation was used for bivariate correlations. The difference between groups was assessed by χ^2^ test (proportions) or Mann–Whitney U-test (asymmetrically distributed variables).

The influence of RBP4 on PSD was performed by univariate and multivariate binary logistic regression analysis. In the multivariate analysis, significant confounding factors which were tested in the univariate analysis were adjusted. Results were expressed as adjusted odds ratios (OR) with the corresponding 95% confidence interval (CI). For a more detailed exploration of the RBP4 and outcome, we also used multivariate analysis models to estimate adjusted OR and 95% CIs of PSD for RBP4 quartiles (with lowest quartile as reference).

Further, a receiver operating characteristic (ROC) curve analysis was used to identify the cut-off point on the serum levels of RBP4 on admission with the greatest sensitivity and specificity to predict poor outcomes at the 3-month follow-up. Area under the curve (AUC) was calculated as measurements of the accuracy of the test. The influence of elevated levels of RBP4 (≥cut-off) on poor outcomes was also performed. Furthermore, to test whether the RBP4 level improves score performance, we considered the two nested logistic regression models with RBP4 and the established risk factors as compared with the established risk factors only.

At last, we conducted analyses separately amongst cases who experienced with and without diabetes. All statistical analysis was performed with SPSS for Windows, version 22.0 (SPSS Inc., Chicago, IL, U.S.A.), the ROCR package (version 1.0-2), and GraphPad Prism 5.0. Statistical significance was defined as *P*<0.05.

## Results

### Patient characteristics

In our study, 299 AIS patients completed 3-month follow-up and were included in the analysis. The median age was 66 (IQR: 55–77) years and 155 (51.8%) were women. In those patients, the median NIHSS score at admission was 9 points (IQR: 5–13). The median time from stroke onset to blood collected was 14.5 (IQR: 8.0–18.0) h. In addition, the number of tissue plasminogen activator-treated patients was 41 (13.7%). Basal characteristics of patients with AIS were provided in Table 1.

**Table 1:** Baseline characteristics of the 299 included stroke patients

Factors	Patients
*n*	299
Age, median (IQR), year	66 (55–77)
Female (sex), *n* (%)	155 (51.8)
BMI, median (IQR), kg/m^2^	28.6 (26.8–30.1)
Infarct volume, median (IQR), ml	14.6 (8.6-20.8)
Temperature, median (IQR), °C	37.2 (36.7–37.5)
Systolic blood pressure, median (IQR), mmHg	143 (130–155)
NIHSS score, median (IQR)	9 (5–13)
Time from onset to blood collection, median (IQR), h	14.5 (8.0–18.0)
Risk factors, *n* (%)	
Hypertension	225 (75.3)
Diabetes mellitus	113 (37.8)
Coronary heart disease	74 (24.7)
Atrial fibrillation	35 (11.7)
Smoking history	48 (16.1)
Family history for stroke and/or CVD	29 (9.7)
Pre-stroke treatment (Yes compared with no)	199 (66.6)
Acute-stroke treatment (rt-PA, Yes compared with no)	41 (13.7)
Causative factors and stroke syndrome	
Small-vessel occlusive	58 (19.4)
Large-vessel occlusive	65 (21.7)
Cardioembolic	105 (35.1)
Other and or unknow	71 (23.8)
TACS	33 (11.0)
PACS	104 (34.8)
LACS	61 (20.4)
POCS	101 (33.8)
Laboratory testing, median (IQR)	
Hs-CRP, mg/dl	0.66 (0.49–1.03)
FBG, mmol/l	6.18 (5.33–6.53)
Triglycerides, mmol/l	1.41 (1.15–1.72)
HDL, mmol/l	1.22 (0.89–1.48)
RBP4, μg/ml	27.3 (18.6–36.4)

### Main results

The serum levels of RBP4 were significantly higher in stroke patients as compared with normal cases (27.3 (IQR: 18.6–36.4) μg/ml compared with 17.6 (IQR: 11.8–23.5) μg/ml; *P*<0.001). Serum levels of RBP4 increased with increasing severity of stroke as defined by the NIHSS score. There was a correlation between levels of RBP4 and NIHSS score (r(Spearman) = 0.437, *P*<0.0001). There was still a significant positive correction between RBP4 serum levels and NIHSS score, using ordered logistic regression after multivariate adjustment for possible confounders (*P*=0.015). Similarly, there was a positive correlation between levels of RBP4 and the infarct volume (r = 0.316, *P*<0.001). In addition, serum levels of RBP4 were also correlated with Hs-CRP (r = 0.254, *P*<0.001), FBG (r = 0.196, *P*=0.001), BMI (R = 0.255, *P*<0.001), and triglycerides (r = 0.225, *P*<0.001). Furthermore, significantly higher RBP4 values were found in women rather than men patients (30.5 (IQR: 21.9–40.1) μg/ml compared with 24.5 (IQR:13.4–32.2) ug/ml; *P*<0.001). We also examined the relationship between serum RBP4 levels and stroke subtypes. The median RBP4 levels were significantly greater for atherosclerosis than for the other stroke subtype groups (32.8 (IQR: 22.8-42.7) μg/ml compared with 25.4 (IQR: 17.5–35.5) μg/ml, respectively; *P*<0.001).

### RBP4 and 3-month functional outcome

**Figure 1:**
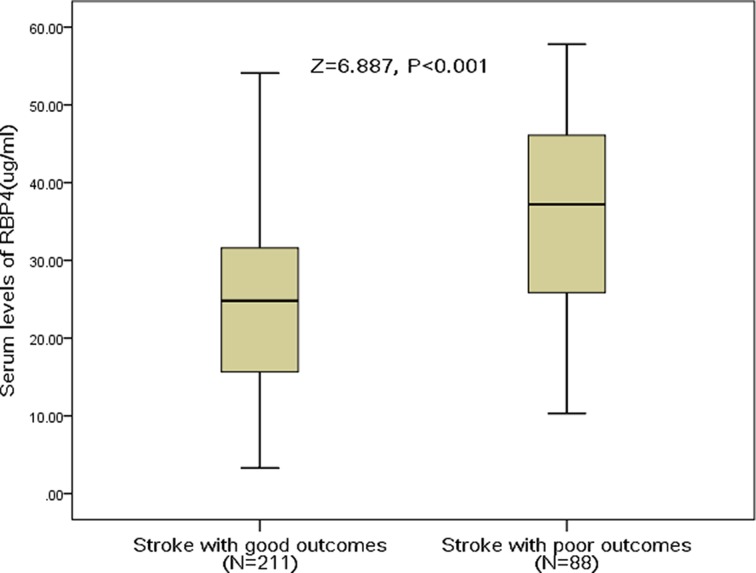
Serum levels of RBP4 in ischemic stroke patients with poor outcomes and good outcomes All data are medians and IQRs; *P*-values refer to Mann–Whitney U-tests for differences between groups. Poor functional outcome was defined as mRS within 3–6.

A poor functional outcome was found in 88 patients (29.4%; 95% CI: 24.3–34.6%) with a median mRS score of 4 (IQR: 3–6). Significantly higher RBP4 values were found in poor outcomes rather than good outcomes’ patients (37.2 (IQR: 25.8–46.2) μg/ml compared with 24.8 (IQR: 15.7–31.8) μg/ml; *P*<0.001; Figure 1). The probability of poor outcomes increased gradually with increasing RBP4 quartiles. The poor outcomes distribution across the RBP4 quartiles ranged between 9.3% (first quartile) and 60.8% (fourth quartile), *P* for trend <0.001.

In univariate logistic regression analysis, we calculated the OR of RBP4 levels as compared with the NIHSS score and other risk factors as presented in Table 2. With an unadjusted OR of 1.18 (95% CI: 1.09–1.30), RBP4 had a strong association with poor functional outcome. After adjusting for all other significant outcome predictors in univariate logistic regression analysis, RBP4 remained an independent outcome predictor with an adjusted OR of 1.09 (95% CI: 1.06–1.13). For a more detailed exploration of the RBP4 and functional outcome relationship, we also used multivariate analysis models to estimate adjusted OR and 95% CIs of poor functional outcome for RBP4 quartiles (with first quartile as reference). In multivariate models comparing the second(Q2), third, and fourth quartiles against the first quartile of the RBP4 (Table 3), RBP4 in Q3 and Q4 were associated with poor functional outcome, and increased risk of poor functional outcome by 144% (OR: 2.44; 95% CI: 1.22–5.03) and 602% (7.02; 3.11–12.24), respectively. The independent association was confirmed using the likelihood ratio test (*P*<0.001).

**Table 2:** Univariate logistic regression analysis for poor outcome

	Univariate analysis	
	OR (95% CI)	*P*
FBG (increase per unit)	1.12 (1.03–1.24)	0.018
RBP4 (increase per unit)	1.18 (1.09–1.30)	<0.001
Hs-CRP (increase per unit)	1.09 (1.04–1.17)	0.009
HDL (increase per unit)	0.93 (0.83–1.03)	0.087
Triglycerides (increase per unit)	1.15 (0.96–1.55)	0.127
Age (increase per unit)	1.08 (1.04–1.15)	0.001
Female sex	1.55 (1.13–2.28)	0.033
BMI (increase per unit)	1.10 (1.03–1.32)	0.021
Infarct volume (increase per unit)	1.20 (1.08–1.33)	0.001
Temperature (increase per unit)	1.02 (0.98–1.09)	0.45
Systolic blood pressure (increase per unit)	0.98 (0.96–1.03)	0.39
NIHSS score (increase per unit)	1.25 (1.11–1.38)	<0.001
Time from onset to blood collection (increase per unit)	1.07 (0.96–1.25)	0.092
Risk factors, yes compared with no		
Hypertension	1.16 (0.92–1.65)	0.19
Diabetes mellitus	1.22 (0.75–1.99)	0.52
Coronary heart disease	1.35 (0.86–2.21)	0.22
Atrial fibrillation	1.76 (1.22–2.58)	0.018
Smoking history	0.62 (0.30–1.17)	0.23
Family history for stroke and/or CVD	1.74 (0.80–3.55)	0.44
Pre-stroke treatment (Yes compared with no)	0.94 (0.83–1.06)	0.072
Acute-stroke treatment (Yes compared with no)	0.88 (0.83–0.95)	0.011
Causative factors and stroke syndrome		
Small-vessel occlusive	0.64 (0.30–1.09)	0.12
Large-vessel occlusive	0.88 (0.60–1.67)	0.69
Cardioembolic	1.13 (0.90–1.55)	0.55
Other and/or unknown	1.22 (0.83–1.93)	0.36
TACS	3.03 (1.55–4.78)	0.017
PACS	0.89 (0.62–1.33)	0.31
LACS	0.74 (0.46–1.36)	0.25
POCS	0.51 (0.26–1.18)	0.20

**Table 3:** Logistic regression model for RBP4 using poor functional outcomes as the dependent variables

Factors	Poor/all, *n* (%)	Univariate logistic regression analysis^2^		Multivariate logistic regression analysis^1^,^2^	
		Unadjusted OR, 95% CI	*P*	Adjusted OR, 95% CI	*P*
RBP4 (increase per unit)	—	1.18 (1.09–1.30)		1.09 (1.06–1.13)	<0.001
RBP4 quartiles^3^					
Q1	7/75, (9.3)	Reference	—	Reference	—
Q2	15/75, (20.0)	2.43 (0.93–6.36)	0.065	1.63 (0.86–3.21)	0.196
Q3	21/75, (28.0)	3.78 (1.50–9.55)	0.003	2.44 (1.22–5.03)	0.015
Q4	45/74, (60.8)	15.07 (6.08–37.5)	<0.001	7.02 (3.11–12.24)	<0.001
RBP4 (cut-off value; ≥ compared with <)	—	8.33 (4.47–15.53)	<0.001	3.58 (1.76–7.75)	<0.001

1 Includes the significant risk factors in univariate logistic regression analysis: age, sex, BMI, infarct volume, NIHSS score, acute treatment, atrial fibrillation, stroke syndrome, and serum levels of Hs-CRP and FBG. Poor functional outcome was defined as mRS within 3–6.

2 The likelihood ratio test, *P*<0.0021.

3 RBP4 in Quartile 1 (<18.6 μg/ml), Quartile 2 (18.6–27.3 μg/ml), Quartile 3 (27.4–36.4 μg/ml), and Quartile 4 (>36.4 μg/ml).

**Figure 2:**
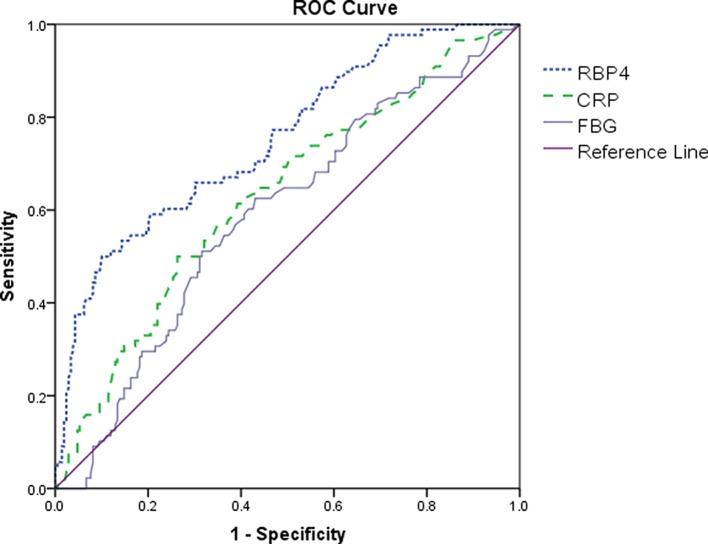
ROC curve demonstrating sensitivity as a function of 1-specificity for predicting the poor outcomes based on the different biomarkers Poor functional outcome was defined as mRS within 3–6.

Based on ROC curves, the optimal cut-off value of serum RBP4 level to diagnose the poor outcomes was 37.4 μg/ml, which yielded the highest sensitivity and specificity (50.0 and 90.0%, respectively; AUC = 0.75, 95% CI: 0.69–0.81; *P*<0.001; Figure 2). With an AUC of 0.75, RBP4 showed a significantly greater discriminatory ability as compared with Hs-CRP (AUC = 0.63; 95% CI: 0.56–0.70; *P*<0.001), FBG (AUC = 0.59; 95% CI, 0.53–0.66; *P*<0.0001), and age (AUC, 0.55; 95% CI, 0.50–0.62; *P*<0.001), while was in the range of NIHSS score (AUC = 0.73; 95% CI: 0.67–0.79; *P*=0.26). Interestingly, RBP4 improved the NIHSS score (AUC of the combined model, 0.79; 95% CI: 0.74–0.85; *P*<0.001). This improvement was stable in an internal five-fold cross-validation that resulted in an average AUC (S.E.M.) of 0.73 (0.032) for the NIHSS and 0.79 (0.028) for the combined model, corresponding to a difference of 0.07 (0.004). In addition, a significant difference in the AUC between the established risk factors alone and the addition of RBP4 concentrations was observed (difference: 0.02 (95% CI: 0.01–0.03); *P*=0.033).

Furthermore, in our study, we found that an increased risk of poor outcomes was associated with RBP4 levels ≥37.4 μg/ml, and increased risk of poor functional outcome by 733% (OR unadjusted: 8.33; 95% CI: 4.47–15.53) and 258% (OR adjusted: 3.58; 1.76–7.75), respectively (Table 3). Interestingly, patients with RBP4 levels ≥37.4 μg/ml was considered as an indicator to predict poor outcomes, the positive predictive value (PPV) and negative predictive value (NPV) were 67.2 and 80.3%, respectively. The diagnostic accordance rate was 77.6%.

### Subgroup analysis

We conducted analyses separately amongst cases who experienced with and without diabetes. Significantly higher RBP4 values were found in diabetic stroke patients (*n*=133) rather than other patients (31.8 (IQR: 21.3–42.5) μg/ml compared with 25.9 (IQR: 16.8–34.9) μg/ml; *P*<0.001). In multivariate logistic regression analysis, we calculated the prognostic value of RBP4 levels in diabetic stroke patients and diabetes-free stroke patients. The data suggested that for each 1 μg/ml increase in serum level of RBP4, the association was stronger amongst diabetic patients (OR = 1.12, 95% CI: 1.08–1.6; *P*<0.001) compared with non-diabetic patients (OR = 1.07, 95% CI: 0.04–1.11; *P*=0.001).

### RBP4 and 3-month mortality

At 3 months, 31 patients (10.4%) had died, RBP4 levels in those patients were more than two times greater as compared with patients who survived (46.8; IQR: 33.7–56.5 μg/ml compared with 23.3; IQR: 15.9–34.9 μg/ml; *P*<0.0001). After adjusting for all other significant outcome predictors (such as age, NIHSS score, and CRP), RBP4 level remained an independent predictor for mortality with an OR of 1.19 (95% CI: 1.08–1.33). We also used multivariate analysis models to estimate adjusted OR and 95% CIs of mortality for RBP4 quartiles (with first quartile as reference). In multivariate models comparing the Q2, Q3, and Q4 against the Q1 of the RBP4, RBP4 in Q2, Q3 and Q4 were associated with mortality, and increased risk of poor functional outcome by 88% (OR: 1.88; 95% CI: 1.21–3.15), 198% (OR: 2.98; 95% CI: 1.45–5.59) and 708% (8.08; 3.39–13.58), respectively.

Based on ROC curves, the optimal cut-off value of serum RBP4 level to diagnose the mortality was 37.5 μg/ml, which yielded the highest sensitivity and specificity (63.8 and 90.9%, respectively; AUC = 0.81, 95% CI: 0.73–0.87; *P*<0.001). With an AUC of 0.81, RBP4 presented a significantly greater discriminatory ability than Hs-CRP (AUC = 0.66; 95% CI: 0.59–0.74; *P*<0.001), age (AUC = 0.60; 95% CI: 0.55–0.66; *P*<0.001), and NIHSS score (AUC = 0.76; 95% CI: 0.71–0.82; *P*=0.03). Similarly, RBP4 improved the NIHSS score (AUC of the combined model = 0.84; 95% CI: 0.78–0.90; *P*<0.001).

## Discussion

Adipose tissue secretes many types of adipokines (such as leptin, interleukin 6, and adiponectin) that play a role in the progression of stroke [24–26]. The present study offers intriguing and possibly important findings of the prognostic role of RBP4 in AIS. The findings of the present study were as following: (i) serum RBP4 levels in patients with AIS were higher compared with controls; (ii) elevated serum levels of RBP4 were powerful biological markers of risk of developing poor functional outcomes even after adjustment by variables. Thus, it may be used as a future therapeutic target in patients with ischemic stroke; (iii) the prognostic value of RBP4 was stronger amongst diabetic patients (OR = 1.12, 95% CI: 1.08–1.6; *P*<0.001) compared with non-diabetic patients (OR = 1.07, 95% CI: 0.04–1.11; *P*=0.001); (iv) RBP4 showed a significantly greater discriminatory ability to predict poor functional outcomes as compared with other biomarkers, such as Hs-CRP, FBG, and age.

Previous studies had suggested that different variables recorded on admission were associated with poor outcome in stroke patients, for example insulin resistance [27], vitamin D deficiency or 25-hydroxyvitamin D [28,29], fatty acid-binding protein 4 [30], C-reactive protein, and homocysteine [31]. However, most of these biomarkers were modest predictors of outcome and unable to improve the prognostic accuracy of the NIHSS. Interestingly, in the present study, we found that RBP4 improved the NIHSS score.

Consistent with our findings, Prentice et al. [32] reported that levels of RBP4 elevated amongst women who develop stroke compared with those who do not develop a stroke. Similarly, patients with CAD had showed elevated RBP4 serum level [3]. However, another study found that elevated levels of RBP4 were not associated with an increased risk of ischemic stroke [33]. In this study, significantly higher RBP4 values were found in poor outcomes rather than good outcomes patients, and elevated serum levels of RBP4 were powerful biological markers of risk of developing poor outcome. Similarly, Liu et al. [34] showed that plasma RBP4 levels were significantly associated with coronary lesion complexity in women with stable CAD and predict incident cardiovascular events, while other studies suggested that elevated serum RBP-4 levels were significantly associated with CAD [35] and CAD severity [3]. However, Liu et al. [36] found that higher RBP4 levels were non-significant associated with a decreased CVD mortality (*P*=0.09) amongst men with type 2 diabetes mellitus in a 22-year prospective study. Different populations, sample sizes, geographical regions, health status, RBP4 ELISA testing kits, and ethnicity may justify the conflicting results observed in those studies.

The possible mechanism of increased levels of RBP4 with poor outcome in acute stroke in patients has not yet established. Two important pathophysiological mechanisms involved in ischemic stroke were inflammation and oxidative stress [37]. Plasma RBP4 levels had been suggested to be associated with an adverse profile of oxidative stress and inflammatory markers in this Chinese population [38]. The following pathways could be considered for the role of RBP4 in the stroke. First, RBP4 has been known as a negative acute phase inflammatory reactant. RBP4 was suggested to induce *in vitro* inflammation in endothelial cells through stimulating expression of proinflammatory molecules [39], and another study showed that RBP4-induced inflammation was largely mediated by TLR4, and in part, through JNK and p38 MAPK signaling [40]. Second, most of the studies reported a positive relation between RBP4 and oxidative stress markers [9] and a negative relation between RBP4 and antioxidant glutathione [41]. Furthermore, cellular oxidative stress leads to the activation of vascular inflammation [42]. Because RBP4 concentrations were positively related to oxidative stress markers, RBP4 may have a role in the initiation of endothelial inflammation. Third, RBP4 levels independently predicted early endothelial dysfunction, linking adipose tissue inflammation and subclinical atherosclerosis in non-diabetic individuals [43]. The inverse relation between RBP4 and flow-mediated vasodilatation (flow-mediated dilation) endothelial dysfunction are evident in normotensive individuals and in diabetic patients [44]. A previous study suggested that epicardial and microvascular coronary endothelial dysfunction independently predict acute cardiovascular events in patients with and without CAD [45]. At last, elevated blood pressure was common during an acute stroke and was associated with unfavorable outcome [46]. Chiba et al. [47] found that increased levels of RBP4 in women were significantly associated with increased levels of systolic blood pressure. Interestingly, lowering RBP4 might reduce blood pressure through enhanced eNOS-mediated vasodilatation and may be a novel therapeutic approach for hypertension [48]. Thus, elevated RBP4 may have a role in stroke prognosis, and its mechanisms need to be elucidated.

Some limitations should be considered. First, the relatively small sample size (*n*=299) and single center might limit the generalization of the results of the present study. Second, without serial measurement of the circulating levels of RBP4, the present study yielded no data regarding when and how long biomarkers were elevated in these patients. In addition, the present study measured RBP4 in serum, not in cerebral spinal fluid (CSF). It was still uncertain whether peripheral RBP4 levels reflect similar changes in the central nervous system (CNS). Third, since the majority of patients with classical cardiovascular risk factors (e.g. diabetes, dyslipidemia, hypertension etc.) were already treated, we cannot rule out the plausible effects of pharmaceutical agents (e.g. statins) on RBP4, leading to underestimation of its predictive power. In addition, the binding of RBP4 to retinol and transthyretin (TTR) was affected by several factors (e.g. serum retinol, vitamin A intake, physical activity, protein-energy malnutrition, and liver and renal diseases) [9]. For studies to succeed, all of these covariates should be considered and adjustments made. However, it was difficult for us to adjust all. Fourth, the use of ELISA kits, with limited dynamic range, could result in conflicting results. Commercial ELISA kits do not differentiate between holo- and apo-RBP4 and evaluate the whole RBP4 concentration. Variability in findings may emerge due to a lack of high affinity and reliable methods for RBP4 measurement [49]. Furthermore, the absence of a standard and stable measurement method to assess blood RBP4 levels restrict the potential clinical use of this marker in the follow-up of ischemic stroke. At last, a major limitation of the present investigation was the cross-sectional design, which prevented us from inferring cause–effect relationship of RBP4 with stroke outcomes. Well-designed longitudinal studies that use larger sample sizes and that consider potential confounders were needed to confirm our observations.

## Conclusion

The data showed that elevated serum levels of RBP4 at admission were associated with severity and prognosis of AIS, suggesting that vitamin A metabolism or impaired insulin signaling could be involved. Further studies should be carried out with respect to what was the cause of the increased serum levels of RBP4 and the role in the pathology of the stroke outcomes.

## Abbreviations

AIS: acute ischemic stroke
AUC: area under the curve
BMI: body mass index
CAD: coronary artery disease
CI: confidence interval
CV: coefficient of variation
CVD: cardiovascular disease
DWI: diffusion-weighted imaging
eNOS: endothelial nitric oxide synthase
FBG: fasting blood glucose
Hs-CRP: high-sensitivity C-reactive protein
IQR: interquartile range
JNK: c-Jun N-terminal kinase
MAPK: mitogen-activated protein kinase
mRS: modified Rankin scale
NIHSS: National Institutes of Health Stroke Scale
OR: odds ratio
PSD: post-stroke depression
RBP4: retinol-binding protein 4
ROC: receiver operating characteristic
TLR-4: Toll-like receptor 4
